# Managing Feline Idiopathic Hypercalcemia With Chia Seeds (*Salvia hispanica* L.): A Case Series

**DOI:** 10.3389/fvets.2020.00421

**Published:** 2020-07-22

**Authors:** Marco Fantinati, Nathalie Priymenko

**Affiliations:** Nutrition Department, École Nationale Vétérinaire de Toulouse, University of Toulouse, Toulouse, France

**Keywords:** hypercalcemia, cats, nutrition, chia seeds, *Salvia hispanica*

## Abstract

**Background:** We describe for the first time the use of chia seeds (*Salvia hispanica* L.) as a non-pharmacological solution in managing feline idiopathic hypercalcemia when dietary change alone fails.

**Case Summary:** Over a 2-year period of time, three female spayed, middle-aged, Domestic Shorthair cats were diagnosed with idiopathic hypercalcemia. Reason for consultation were lethargy and dysorexia, with a single episode of vomiting described in one cat and dysuria in another. Thorough diagnostic work-up included complete blood count, serum biochemistry, urinalysis, ionized calcium, calcemic hormones, parathyroid hormone-related protein, and imaging of chest and abdomen. Based on different nutritional reasons, each cat was switched to a different high-moisture pet food as first-step in managing the disorder: a high-fiber diet, a diet formulated for chronic kidney disease management and a diet designed to prevent calcium oxalate urolithiasis. In the three cats, 6 weeks of dietary change alone did not result in normocalcemia. Before resorting to any pharmacological solution, supplementation to the diet of chia seeds (2 g/cat/day) was started. After 4 weeks from the introduction of *Salvia hispanica* L., all cats achieved normalization of ionized calcium concentration.

**Conclusion:** Chia seeds (*Salvia hispanica* L.) supplementation could be a useful tool in managing feline idiopathic hypercalcemia.

## Introduction

Hypercalcemia in cats may be associated with a variety of conditions which can be either parathyroid-dependent or, as in the majority of cases, parathyroid-independent (1). Between the latter, idiopathic hypercalcemia is the one diagnosis considered exclusive of the feline species, and the diagnosis of which is made *per exclusionem* (2).

According to a review by de Brito Galvão and colleagues, idiopathic hypercalcemia should be addressed as main cause of hypercalcemia in cats (2), while recent analysis on the Cornell University's database (2007-2017) suggests that malignancy and renal failure are more common causes (3). Regardless of which place claims on the podium, being first or third in terms of frequency, it is clear that idiopathic hypercalcemia is one of the important diagnoses of hypercalcemia in cats.

Despite the etiopathogenesis is still not known, explanations for the development and maintenance of increased circulating calcium levels lie in a metabolic disorder of this mineral: enhanced bone resorption, augmented intestinal absorption, decreased renal excretion, or a combination of these (4).

Cats diagnosed with idiopathic hypercalcemia are typically middle-aged (mean, 5.8–9.8 years), with both sexes equally represented (5, 6). Cats can be frequently asymptomatic (6), but when it is not the case, depending on literature source, either anorexia (2) or vomiting (5) are the most common signs, with lethargy, weight loss, constipation, dysuria, hematuria, polyuria, and polydipsia as other occurring symptoms (2, 5, 6).

To rule out the long list of potential causes of hypercalcemia, patient evaluation should start with a thorough history-taking and physical examination, followed by a minimum database comprising complete blood count, serum biochemistry, ionized calcium (iCa), PTH-measurement, urinalysis, imaging of chest and abdomen (radiography and ultrasonography) (7). Extended database may include serum 25-hydroxycholecalciferol (calcidiol), 1,25-dihydroxycholecalciferol (calcitriol), 24,25-dihydroxycholecalciferol, concentrations of parathyroid hormone-related protein (PTHrP), ultrasonography of the neck, or exploratory surgery (2).

A typical cat with idiopathic hypercalcemia exhibits mild to moderate increases in total (tCa >2.75 mmol/L) and ionized (iCa >1.40 mmol/L) calcium serum levels (1), with normal serum albumin and usually accompanied by concentrations of calcemic hormones (parathyroid hormone and vitamin D metabolites) which are either low or perfectly within laboratory reference range (1, 2).

Dietary change is considered the first-line treatment in the management of the disease (2). Lack of published prospective studies, and conflicting results (5, 8), make it currently not possible to state which nutritional aspect is the most significant in the regulation of calcium homeostasis in this pathology.

High-fiber diets can supposedly decrease intestinal calcium absorption by affecting transit time and by binding dietary calcium into the gut's lumen (5, 8). Diets formulated for chronic kidney disease management are usually restricted in calcium, and thanks to their less acidifying power can potentially decrease calcium release from bones, a combination which theoretically can affect circulating ionized calcium (5, 9). Finally, diets designed to prevent calcium oxalate urolithiasis have also been used, mainly for their calcium restriction, and because of their proven effect in lowering both concentration and excretion of calcium in urine (10).

When these non-pharmacologic solutions fail in restoring normocalcemia, two pharmacologic treatments can be pursued. Historically, first treatment choice was glucocorticoids, which can decrease calcium concentration via multiple mechanisms, such as decreased intestinal absorption, decreased renal reabsorption, and decreased bone mineral mobilization (11). Today, bisphosphonates have become a safer alternative, with oral alendronate as treatment of choice (12). Mechanism of action is the inhibition of osteoclastic bone resorption by down-regulation of the enzyme farnesyl diphosphate synthase in the mevalonate pathway. Produced metabolites are required for intracellular signaling, as well as hydroxyapatite crystal dissolution. Moreover, metabolism of bisphosphonates activates an intracellular ATP analog which is believed to induce apoptosis of osteoclasts (13).

The present case report, explores the use of chia seeds as alternative non-pharmacologic solution in the treatment of feline idiopathic hypercalcemia.

Native to southern Mexico and northern Guatemala, Chia (*Salvia hispanica* L.) is a summer annual herbaceous plant of the Lamiacae family (14). In recent years, its seeds have drawn the attention of the scientific community because of their high nutritional and functional value (14–18). Chemical composition of chia seed has been analyzed by many researchers and reviewed by the European Food Safety Authority (16, 19–22). Results highlighted a high fat (18–39% as fed) and protein (15–26% as fed) content, making *Salvia hispanica* the richest botanical source of α-linoleic acid (ALA), and an important protein source too, having a greater protein concentration in comparison to cereals (16, 23). Fiber content is also high, with 100 g of whole chia seeds containing 34–40 g of total dietary fiber, of which 85–93% insoluble and 7–15% soluble fiber, respectively (17, 20). Chia seeds are also rich in macrominerals (calcium, phosphorus, magnesium, potassium) (14, 16) and can be a source of trace minerals (iron and zinc) (24), fat-soluble (A, D, E, and K), and water-soluble (C, thiamine, riboflavin, and niacin) vitamins (25). Furthermore, other active compounds such as phenolic acid derivatives and flavonoids have been identified, with interesting amounts of rosmarinic acid, caffeic acid, chlorogenic acid, kaempferol, and daidzein (21).

Due to the nutritional profile rich in bioactive components (e.g., fiber, omega-3 fatty acids), multiple studies have explored in laboratory animals and humans the use of this food as a supplement in the treatment of several disorders such as obesity, cardiovascular disease, inflammatory-related disorders, hypertension, hyperlipidemia, cancer, oxidative stress, diabetes, and others (26–28).

To the author's knowledge there is no published literature on supplementation of chia seeds in feline nutrition. Furthermore, nothing has yet been published on its use in the treatment of feline idiopathic hypercalcemia.

Based on literature, active components present in chia seed, may have possible influences toward some of the supposed underlying mechanisms of this disease pathogenesis, such as increasing intestinal binding of calcium and decreasing bone resorption (24, 29, 30). For this reason, chia seed supplementation has been tested in three cats diagnosed with idiopathic hypercalcemia, after dietary change alone did not lead to normocalcemia.

## Case Description

Over a 2-year period (2018-2020), a diagnosis of idiopathic hypercalcemia was concluded in three cats after a thorough diagnostic process. Reason for consultation were lethargy and dysorexia, with a single episode of vomiting described in one cat and dysuria in another. All three were female spayed middle-aged (range 5.8–8.2 years) Domestic Shorthair cats, lived strictly indoor, were regularly dewormed and vaccinated with core vaccines.

### Diagnostic Approach

Diagnostic work-up followed the same criteria for the three patients, starting with careful history-taking and physical examination, continuing with blood testing and imaging.

Medical history ruled out the ingestion of a cholecalciferol rodenticide. No relevant clinical findings were found during routine physicals. Blood sampling was performed after a 12-h fast. Complete blood count and serum biochemistry revealed a slight increase of total calcium paralleled by normal serum protein profile. Hypercalcemia was confirmed by increased levels of ionized calcium (>1.40 mmol/L) (1). Ionized calcium concentration was measured on anaerobically-collected serum samples using an ion-selective electrode (VetStat™, IDEXX Laboratories Inc., Westbrook, Maine). Serum calcemic hormones (intact PTH, calcidiol and calcitriol) and PTHrP measurement were within reference intervals. Lateral and ventrodorsal radiography of chest and abdomen, followed by abdominal ultrasonography did not show any abnormality. No radiopaque calcium oxalate (CaOx) uroliths were visible in the urinary system, but urinalysis showed CaOx crystals with a 5.8 urine pH in the dysuric cat. Case characteristics and main results of diagnostics are summarized in Table 1.

**Table 1:** Signalment, symptoms and main diagnostics' results.

	**Cat A**	**Cat B**	**Cat C**	
Breed	DSH	DSH	DSH	
Sex	Fs	Fs	Fs	
Age (years)	6.5	5.8	8.2	
Clinical signs	lethargy, dysorexia, vomiting	lethargy, dysorexia, dysuria	lethargy, dysorexia	
**Parameter**				**Reference Interval**
iCa (mmol/L)	1.7	1.6	1.6	1.1–1.4
tCa (mmol/L)	2.83	2.70	2.75	2.0–2.6
Albumin (g/L)	35.4	37.2	35.8	27–39
Total protein (g/L)	68.7	70.1	58.9	55–71
Intact PTH (pmol/L)	0.8	1.5	1.2	0–4.0
Calcidiol (nmol/L)	102.4	87.6	113.8	65–170
Calcitriol (pmol/L)	52.3	58.1	48.7	50–100
PTHrP (pmol/L)	–	–	–	<1.0
Imaging^*^	–	–	–	NA
SDMA (μg/dL)	8.3	6.5	11.1	<15.0
Creatinine (μmol/L)	91.5	88.7	102.5	80–229
BUN (mmol/L)	7.5	6.9	8.1	5.4–10.4
USG	1.040	1.045	1.035	NA
Urine pH	6.3	5.8	6.4	NA
UP/C	<0.2	<0.2	<0.2	<0.2
CaOx crystals	–	+	–	NA

* *radiography chest and abdomen, ultrasonography abdomen*.

*DSH, Domestic Shorthair; Fs, female spayed; NA, Not Applicable; iCa, ionized calcium; tCa, total calcium; PTH, parathyroid hormone; PTHrP, parathyroid hormone-related protein; SDMA, symmetric dimethylarginine; BUN, blood urea nitrogen; USG, urinary specific gravity; UP/C, urine protein to creatinine ratio; CaOx, calcium oxalate*.

### Nutritional Assessment

All cats were fed a dry pet food on a meal-feeding regimen. According to owners, the amount fed kept their cats' body weight stable. To estimate energy food intake, metabolizable energy of each pet food was determined via NRC (2006) predictive equation using crude fiber (31). Body weight was measured for each cat, and body fat was assessed via body condition scoring (BCS) on a 9-point scale (32). Body condition assessment was completed by evaluation of lean tissue via muscle mass scoring (MMS) on a 4-point scale (33). Pet food characteristics and details according to patient are summarized in Table 2.

**Table 2:** Energy intake, body condition assessment, and pet food characteristics at the time of diagnosis.

	**Cat A**		**Cat B**		**Cat C**	
BW (kg)	4.4		4.7		5.2	
BCS (9-point scale)	5		6		6	
MMS (4-point scale)	3		3		3	
Ideal BW (kg)	4.4		4.2		4.7	
Energy Intake	0.91 MJ/day	218 kcal/day	1.10 MJ/day	262 kcal/day	1.17 MJ/day	279 kcal/day
**Dry Pet Food**	**MJ/kg**	**kcal/kg**	**MJ/kg**	**kcal/kg**	**MJ/kg**	**kcal/kg**
ME	15.18	3629	15.67	3745	16.21	3874
	**g/MJ**	**g/Mcal**	**g/MJ**	**g/Mcal**	**g/MJ**	**g/Mcal**
Crude Protein	24.37	101.96	17.23	72.10	18.51	77.44
Crude Fat	7.90	33.07	8.30	34.71	9.25	38.72
Crude Fiber	4.08	17.08	2.55	10.68	3.02	12.65
Ca	0.72	3.03	0.70	2.94	0.60	2.53
P	0.66	2.76	0.54	2.24	0.40	1.68
Na	0.40	1.65	0.32	1.34	0.19	0.77
Mg	0.06	0.25	0.05	0.21	0.04	0.18
Omega-3	0.39	1.63	0.40	1.68	0.67	2.81
Omega-6	1.80	7.55	2.13	8.89	2.37	9.91
n-6/n-3 ratio	4.6:1		5.3:1		3.5:1	

*To compare diets, nutrients are presented according to energy density*.

*BW, body weight; BCS, body condition score; MMS, muscle mass score; ME, metabolizable energy; Ca, calcium; P, phosphorus; Na, sodium; Mg, magnesium*.

### Dietary Change

According to literature, dietary change is the first step in managing feline idiopathic hypercalcemia for non-clinical or minimally symptomatic cats (1, 2, 7). High-moisture pet foods are recommended to promote urine dilution and prevent CaOx urolithiasis (34). There is not a consensus on the best diet for the treatment of feline idiopathic hypercalcemia. In the present study were prescribed the same diets that for different nutritional reasons have been used in treating this condition in literature (5, 8).

To avoid diet-induced microbiota's perturbation, for each of the study's cats it was performed a 7-day dietary transition to a high-moisture pet food. Cat A received a high-fiber diet (Hill's™ Prescription diet™ w/d), cat B a urinary care diet (Hill's™ Prescription diet™ c/d), and cat C a kidney care diet (Hill's™ Prescription diet™ k/d). Daily amount was calculated on the same energy intake already known to preserve a stable body weight. For the purpose of the study, it was decided to not decrease the calorie intake in the two slightly overweight cats.

Recheck of serum ionized calcium levels was performed after 6 weeks of diet change. None of the cats achieved normocalcemia during this period. However, owners reported good acceptance of the high-moisture diet, which at least had positive effects diluting urines and balancing urine pH. Cats were still lethargic. Results of serum ionized calcium and characteristics of the prescribed diets are detailed in Table 3.

**Table 3:** Prescribed high-moisture pet foods' characteristics with data between parentheses indicating the amount of nutrient according to daily food intake.

	**Cat A**		**Cat B**		**Cat C**		**Chia**
**Pet Food**	Hill's™ w/d		Hill's™ c/d		Hill's™ k/d		2 g/cat/d
	**MJ/kg**	**kcal/kg**	**MJ/kg**	**kcal/kg**	**MJ/kg**	**kcal/kg**	**MJ**
ME	3.59 (0.91)	857 (218)	4.80 (1.10)	1148 (262)	5.46 (1.17)	1304 (279)	0.04
	**g/MJ**	**g/Mcal**	**g/MJ**	**g/Mcal**	**g/MJ**	**g/Mcal**	**g**
Crude Protein	25.94 (23.63)	108.52	22.07 (24.21)	92.33	15.76 (18.40)	65.95	0.42
Crude Fat	9.20 (8.38)	38.51	11.66 (12.79)	48.78	12.10 (14.12)	50.61	0.66
Crude Fiber	9.20 (8.38)	38.51	0.62 (0.69)	2.61	1.28 (1.50)	5.37	0.48
Ca	0.64 (0.58)	2.68	0.42 (0.46)	1.74	0.44 (0.51)	1.84	0.01
P	0.45 (0.41)	1.87	0.35 (0.39)	1.48	0.26 (0.30)	1.07	0.02
Na	0.25 (0.23)	1.05	0.19 (0.21)	0.78	0.13 (0.15)	0.54	0.02
Mg	0.04 (0.04)	0.18	0.04 (0.04)	0.16	0.04 (0.04)	0.15	0.01
Omega-3	0.17 (0.15)	0.70	0.46 (0.50)	1.92	0.53 (0.62)	2.22	0.40
Omega-6	1.73 (1.58)	7.23	2.23 (2.44)	9.32	2.00 (2.33)	8.36	0.10
n-6/n-3 ratio	10.0:1	4.5:1	3.8:1	0.3:1			
**Follow-up** (6 weeks)							
iCa (mmol/L)^*^	1.6		1.7		1.6		

*Details of nutrient added per day by chia supplementation are displayed in the right column. Ionized calcium at 6-weeks is shown in the lower lines*.

* *Reference interval is 1.1–1.4 mmol/L*.

*ME, metabolizable energy; Ca, calcium; P, phosphorus; Na, sodium; Mg, magnesium; iCa, ionized calcium*.

### Chia (*Salvia hispanica* L.) Seed Supplementation

Chia (*Salvia hispanica* L.) seed supplementation into the diet was started at a dose of 2 g/cat/day, considering that human medicine literature usually suggests 25 g/person/day (for an average person of 70 kg BW) (20, 35). Before mixing chia to the first morning meal, owners were instructed to soak seeds in 20 mL of water for 20 min to allow mucilage formation because of gum's exudation from the seed outer layer (36).

Supplementation was continued once a day for 4 weeks before ionized calcium recheck. Contribution to the diet of chia seed supplement is detailed in Table 3. Whole chia seed basic composition and chemical constituents are summarized in Table 4.

**Table 4:** Nutritional mean value of *Salvia hispanica* L. seeds (14, 20–22, 24).

**Energy**	**MJ/100 g**	Dietary fiber^*^	**g/100 g**
Energy	2,03	Insoluble Fiber	34.67
**Nutrient**	g/100 g	Soluble Fiber	4.01
Dry matter	92.2	Antinutritional factor^*^	g/100 g
Protein	21.1	Phytic Acid	0.71
Fat	32.8	**Essential amino acids**	g/100 g
Carbohydrate	37.5	Arginine	2.14
Crude Fiber	24.0	Histidine	0.53
Ash	4.6	Isoleucine	0.8
n-6/n-3 ratio	1:3	Leucine	1.37
**Mineral**	mg/100 g	Lysine	0.97
Sodium	0.94	Methionine	0.59
Potassium	667	Phenylalanine	1.02
Calcium	557	Threonine	0.71
Iron	6.3	Tryptophan	0.44
Magnesium	380	Valine	0.95
Phosphorus	751	**Non-essential amino acids**	g/100 g
Zinc	4.46	Cystine	0.41
Copper	1.93	Tyrosine	0.56
Selenium	0.006	Alanine	1.04
Vitamin	mg/100 g	Aspartic acid	1.69
Vitamin A	0.05	Glutamic acid	3.50
Vitamin C	<3.0	Glycine	0.94
Vitamin E	0.74	Proline	0.78
Thiamine	0.18	Serine	1.05
Riboflavin	0.04	**Fatty acid profile**	% on the lipid fraction
Niacin	6.13	**Polyunsaturated fatty acids**	%
Pyridoxine	0.1	Linoleic acid	18.89
Folate	0.05	Linolenic acid	63.79
**Polyphenols**	μg/100 g	**Saturated fatty acids**	%
Caffeic acid	27	Myristic acid	0.06
Ferulic acid	traces	Pentadecanoic acid	0.04
Chlorogenic acid	4	Palmitic acid	7.1
Rosmarinic acid	927	Margaric acid	0.06
Myricetin	118	Stearic acid	3.24
Quercetin	0.17	Arachidic acid	0.24
Kaempferol	0.013	Behenic acid	0.08
**Flavonoids**	μg/100 g	Lignoceric acid	0.1
Daidzin	6.6	**Monounsaturated fatty acids**	%
Glycitin	1.4	Palmitoleic acid	0.2
Glycitein	0.5	Margaric acid	0.06
Genistin	3.4	Oleic acid	10.53
Genistein	5.1	Eicosenoic acid	0.16

* *Concentration of dietary fiber and phytic acid on chia flour*.

### Outcome

Follow-up at 4 weeks showed normalization of serum ionized calcium in all three cats, regardless of the previously prescribed diet (Cat A, iCa 1.2 mmol/L; Cat B, iCa 1.3 mmol/L; Cat C, 1.2 mmol/L). Owners also reported a more active behavior without other symptoms.

Recheck after another 6 weeks (10 weeks since the introduction of chia in the diet) confirmed normocalcemia (Cat A, iCa 1.1 mmol/L; Cat B, iCa 1.2 mmol/L; Cat C, 1.2 mmol/L).

## Discussion

This case report describes chia seed effects in managing feline idiopathic hypercalcemia. All three cats, which failed to achieve normocalcemia with dietary change alone, showed physiological levels of ionized calcium after 4 weeks of chia supplementation regardless of diet type.

Despite the lack of published literature on the use of chia seeds in managing feline idiopathic hypercalcemia and in general on its supplementation in cats' diets, an in depth analysis of this food composition can offer some explanation on its possible effects on calcium metabolism.

It is still controversial if cats with iCa concentration slightly above reference should be treated or not, as they usually do not manifest any symptom. In fact, idiopathic hypercalcemia can often be an incidental finding during pre-operative blood analysis and in some cases can resolve without any treatment. Nonetheless, it is widely accepted that if ionized hypercalcemia persists (or increases) and mild clinical signs are present, first-line dietary treatment should be initiated.

High-fiber diets have already been used for achieving normocalcemia with conflicting results (5, 8). In a study by McClain and colleagues, four cats received a diet rich in insoluble fiber (Hill's™ Prescription diet™ w/d) while a fifth cat was supplemented with psyllium, a food known for its high concentration of soluble fiber. Hypercalcemia was resolved in all cats based on total calcium levels (8). Another study, using the same diet high in insoluble fiber, did not show any beneficial effect on ionized calcium in three cats diagnosed with idiopathic hypercalcemia (5). Searched effects were an increased binding of intestinal calcium, and a modified intestinal transit time, to prevent and reduce calcium absorption, respectively (37, 38).

Chia seeds are an important source of dietary fiber (34–40 g/100 g), rich in both insoluble (85–93%), and soluble (7–15%) fiber (17, 20), therefore contributing to the total amount of fiber in the diet. Furthermore, soaking chia seed in water triggers its gelation process, with exudation from the seed's coat of a transparent mucilaginous gel (chia mucilage) composed essentially of soluble fiber (gum), easing this nutrient's effects on transit time, digestion and fermentation (36, 39). In the present study, supplemented chia seed brought to a 5–6% increase of the crude fiber concentration in all three diets.

Effects on calcium binding and transit time will be related only to the meal in which moistened chia seeds are supplemented, making doubtful that the aforementioned effects alone are responsible for normalization of ionized calcium levels, considering also that normocalcemia was achieved in all cats where the contribution to the daily amount of dietary fiber varies greatly between the three diets.

Dietary restriction of calcium is considered important in managing idiopathic hypercalcemia. Recommendations start with a calcium level in the diet at least below 0.48 g/MJ (<2 g/Mcal), lowering to less than 0.14 g/MJ (<0.6 g/Mcal) when normocalcemia is not achieved (2).

Two of the three diets used had a calcium level below initial recommendation, but this alone was not sufficient to normalize ionized calcium. High-moisture diets have higher calcium contents because of their lower energy density, therefore, lowering calcium to less than 0.14 g/MJ was not possible due to managing choices in the study.

Paradoxically, chia seeds are a food relatively rich in calcium (557 mg/100 g) (22). Despite being so, calcium present in chia seeds is not well-absorbed, metabolized, and/or utilized by the organism (30). This reduced bioavailability has been proven in Wistar rats, and explained by the increased fecal excretion of calcium caused by chia supplementation and its richness in phenolic compounds, tannins, and phytate which can increase calcium binding, thus prevent its absorption (30). As total dietary fiber increases, digestibility decreases, paralleled by increased excretion of fecal dry matter. It has been proven in dogs that fecal dry matter excretion, which is higher when additional fiber is supplemented to the diet, strongly impacts fecal calcium excretion (38).

All diets used in the present study had high omega-6 to omega-3 fatty acids ratio (n-6/n-3 ratio). On the contrary, chia seeds, if compared to other botanical sources, contain the highest known amount of α-linolenic acid (omega-3), with an omega-6 to omega-3 ratio of 1:3 (0.3–0.35) (14). Chia seed supplement (2 g/cat/day) contributed to the dietary pool of fatty acids bringing ~0.4 g of omega-3 and 0.1 g of omega-6, which may seem negligible but is enough to positively affect the n-6/n-3 ratio. The latter decreased from 10.0:1 to 3.3:1 in Cat A, 4.5:1 to 3.0:1 and 3.8:1 to 2.5:1 in Cats B and C, respectively. Increasing dietary omega-3 has been shown to have positive effects on inflammation, oxidative stress and several disorders both in human and veterinary medicine (40). Consumption of chia seeds in rats led to inhibition of inflammatory cytokines, lowering the inflammatory state (30), a phenomenon which could be ascribable to its richness in omega-3 and other bioactive compounds such as polyphenols and flavonoids (20). Overproduction of pro-inflammatory cytokines, has been proven to enhance bone resorption, via activation of osteoclasts and inhibition of osteoblasts (41). Therefore, anti-inflammatory effects of chia seed supplementation could positively affect calcium metabolism, making for another contribution in normalization of ionized calcium.

Long-term dietary intake of chia seeds has been associated with increased bone health in Sprague-Dawley rats (42). High intake of omega-3 has in fact growing evidence of affecting bone metabolism. Mechanisms allowing fatty acids to reach bone cells are still partly a conundrum, but *in vitro* studies confirm the aforementioned downregulation of osteoclastic activity (43).

*Salvia hispanica* L. richness and variety in phenolic acid derivatives and flavonoids (21) could also influence calcium metabolism. Plant polyphenols have positive effects in reducing oxidative stress, which acts by enhancing bone resorption, differentiation, and activity of osteoclasts (44). For example, rosmarinic acid, which is one of the most represented polyphenols in chia seeds (927 μg/100 g) has proven effects on inhibiting osteoclastic differentiation (45). Similar mechanisms can also be promoted by flavonoids, which act on cell signaling pathways influencing osteoclastic differentiation, therefore inhibiting bone resorption (46). However, it should be said that the concentration of these metabolites found in the amount of chia seeds supplemented in the present study, is very small if compared to what experimentally used to achieve biological effects.

Overall, several bioactive compounds can potentially inhibit bone resorption by acting on osteoclasts which are the only cells capable of this role (25, 29, 30, 44–46). It is plausible that such mechanism plays an important role in managing feline idiopathic hypercalcemia. In fact, therapy with oral nitrogen-containing bisphosphonates (e.g., alendronate) is considered the first-line treatment when dietary change alone fails to normalize serum ionized calcium levels (12) and these drugs effectiveness relays on a potent inhibition of bone resorption (12, 13).

Several nutrients present in chia seeds, such as omega-3 fatty acids, soluble and insoluble fibers, polyphenols, and flavonoids, can affect calcium metabolism (25, 29, 30, 44–46). It is reasonable to say that normalization of ionized calcium concentrations in feline idiopathic hypercalcemia is ascribable not to one single nutrient, but more seemingly to the combined effects of all these bioactive compounds.

## Conclusion

Supplementation with chia seeds was successful in improving symptoms and normalizing serum ionized calcium levels in cats with idiopathic hypercalcemia, when previous dietary change was ineffective. Normocalcemia was achieved in 4 weeks of supplementation regardless of diet type. Ten weeks after the beginning of daily chia seed supplementation, ionized calcium was still in its physiological range. Despite the promising outcome, absence of control group and small number of cats are important limitations of the present work. A randomized controlled trial with a larger population will be necessary before the routine supplementation of chia seeds can be recommended in feline idiopathic hypercalcemia.

## Data Availability Statement

All datasets presented in this study are included in the article/supplementary material.

## Ethics Statement

Only client-owned animals were involved in the present study. University's veterinary counseling terms were signed by every owner prior to nutritional consultations and communication with owners ensured that they had enough information about the care plan. Informed consent was freely granted by each of the participants.

## Author Contributions

MF personally followed all presented cases, wrote the manuscript, and literature review. NP gave birth to the idea, contributed by giving intellectual mentorship and by reviewing the manuscript final draft. All authors contributed to the article and approved the submitted version.

## Conflict of Interest

The authors declare that the research was conducted in the absence of any commercial or financial relationships that could be construed as a potential conflict of interest.
